# Predicting memorability of face photographs with deep neural networks

**DOI:** 10.1038/s41598-023-49904-6

**Published:** 2024-01-13

**Authors:** Mohammad Younesi, Yalda Mohsenzadeh

**Affiliations:** 1 Department of Computer Science, Western University, London, ON Canada; 2 Western Institute for Neuroscience, Western University, London, ON Canada; 3 Vector Institute for Artificial Intelligence, Toronto, ON Canada

**Keywords:** Human behaviour, Computational models, Computer science

## Abstract

With the advent of social media in our daily life, we are exposed to a plethora of images, particularly face photographs, every day. Recent behavioural studies have shown that some of these photographs stick in the mind better than others. Previous research have shown that memorability is an intrinsic property of an image, hence the memorability of an image can be computed from that image. Moreover, various works found that the memorability of an image is highly consistent across people and also over time. Recently, researchers employed deep neural networks to predict image memorability. Here, we show although those models perform well on scene and object images, they perform poorly on photographs of human faces. We demonstrate and explain why generic memorability models do not result in an acceptable performance on face photographs and propose seven different models to estimate the memorability of face images. In addition, we show that these models outperform the previous classical methods, which were used for predicting face memorability.

Every day, we meet new people or encounter new faces on social media. Some of these faces stick in our minds, while others are forgotten quickly. Image memorability is the probability that an observer will detect the repetition of an image after a single exposure to that image when presented amidst a stream of images^1^. Despite individual differences in remembering visual events^2,3^, it has been shown that the image’s memorability is consistent across people and over various time lags^1,4–10^. In essence, people show consistent behavior in remembering some images and forgetting others. These findings have led to the idea of predicting an image’s memorability based solely on the image itself, estimating what images are more or less memorable than others^11^.

Previous studies have indicated that individuals fail to accurately predict the memorability of images^5^. Research has found that images that are standing out from their context are more likely to be remembered^12^. Additionaly, distinctiveness plays a key role in face recognition, i.e., distinctive faces are recognized better than typical ones^13^. Furthermore, faces perceived as unusual in appearance have been shown to be remembered better than those considered typical^14–16^.

Bainbridge et al.^4^ investigated what factors are contributing to face memorability, examining the role of twenty personalities (e.g. interesting/boring and calm/aggressive), social, and memory-related traits. After running a multiple linear regression model on these face attributes and memorability scores, they found that the combination of these attributes can only explain a small portion of the variance of the memorability scores. This suggests that the memorability of an image depends on the image itself, rather than on a limited set of identifiable attributes. It is worth highlighting that the concept of face memorability pertains to the memorability of a facial photograph, rather than an individual’s actual visage (i.e. a photograph of Tom Cruise could be more memorable than another photograph of his). Indeed, in our recent work^17^, we developed a method to control and modify the memorability of a face photograph by photo-editing techniques based on generative models.

Various studies have aimed to predict image memorability. One of the earliest methods was proposed by Khosla et al.^18^, which used dense global features such as HOG ^19^ and SIFT^20^ for predicting face memorability. However, these methods were not fully automatic and required manual tuning. Convolutional neural networks^21^ have shown great performance in image classification task^22^ and since then have been used in various computer vision and machine learning tasks. Khosla et al.^11^ introduced the first model that used a convolutional neural network model called *MemNet* for predicting image memorability. It was trained by fine-tuning Hybrid-CNN^23^ and performed near human consistency in rank correlation.

Most recent research has focused on improving the performance of these models by employing attention mechanisms^24^ and residual blocks^25^. Lu et al.^26^ tried to find out what are the elements that make outdoor natural scenes memorable. They discovered combining high-level features of scene category and deep features can result in improving the model performance in predicting memorability. While memorability is an intrinsic feature of an image, some works studied the extrinsic effects such as eye movements in predicting the memorability^12^.

We see people’s faces in different conditions e.g. while they are happy, angry, or neutral. Bainbridge et al.^27^ shed light on how memorability changes with different transformations of the human face (neutral, happy, angry, 3/4 view, and profile view). They found that memorability is highly consistent within each image transformation as well. It means regardless of the person’s face being neutral or happy, if she has got a memorable face, we’ll remember her face and vice versa.

In this work, we have focused on predicting the memorability of face photographs. As Squalli-Houssaini et al.^28^ have demonstrated, deep neural network models (including MemNet^11^ and other memorability networks that are trained on LaMem data set) succeed in predicting the memorability of scene and objects images. Here, we evaluated several memorability models (including original MemNet) which are trained on LaMem dataset on predicting memorability of face images. Consistent with Squalli-Houssaini et al.^28^, our results demonstrate that these memorability models fail in predicting the memorability of face photographs. Then, using 10k US Adult Faces Database^4^, we fine-tuned VGG16^29^, ResNet50^30^ and SENet50^31^ which are pre-trained on VGGFace data set^32^ for a face identification task to predict the face memorability scores. We also fine-tuned MemNet which is trained and performs well on LaMem^11^ data set to predict face memorability. We hypothesize that the models which are pre-trained on face recognition task and then fine-tuned on face memorability prediction task outperform those which are pre-trained on LaMem and then fine-tuned for the face memorability prediction. The main reason is that models pre-trained on face images for a face recognition task will be more efficient in extracting face features that later can be utilized for predicting face memorability scores. Our proposed models outperformed the previous model^18^ and got close to human consistency correlation in predicting face memorability.

## Results

This section presents the key findings and discoveries obtained from the experimental analysis of training models for face photographs memorability prediction. First, we demonstarte that the current memorability models that are trained on LaMem fail in predicting face memorability scores. Then, we propose new architectures for predicting face memorability scores. These models perform close to human consistency in rank correlation. At the end, we showed that the trained deep models, can be used in predicting memorability scores of both oval-shaped and square-shaped face images.

### State-of-the-art memorability models fail to predict face memorability

We evaluated three state-of-the-art memorability models which are trained on LaMem dataset on the task of predicting face memorability. For this, we used 10k US Adult Faces Database^4^ which contains 10,168 natural face photographs. 2222 of these faces were annotated with memorability scores. All the faces in this dataset are oval-shaped with the same height of 256 pixels but variable widths.

**Figure 1:**
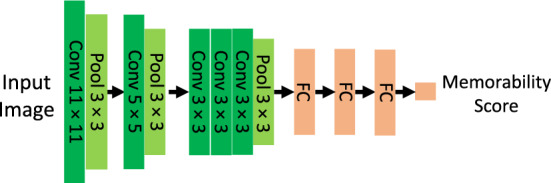
Architecture of MemNet.

**Figure 2:**
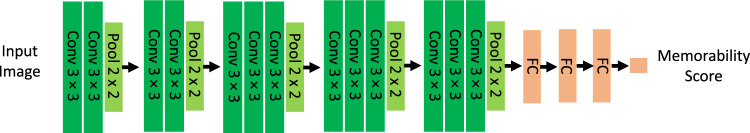
Architecture of MemVGG.

**Figure 3:**
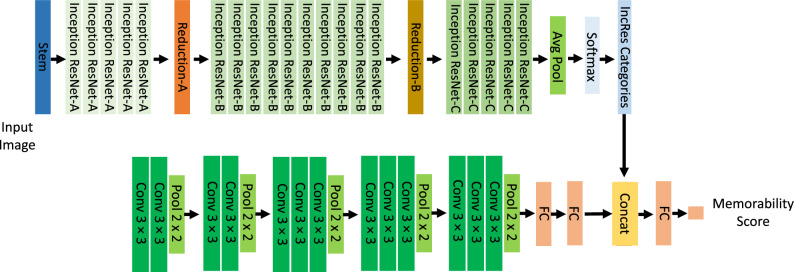
Architecture of IncResMem. The input image goes through two parallel streams and at last stage, the extracted features are combined to predict the memorability.

We tested MemNet on the task of predicting memorability scores of the 10k US Adult Faces Database. Figure 1 depicts the architecture of the MemNet. The original MemNet is an old version and only available on Caffe. Therefore, we followed the methods described in the paper and retrained the model in PyTorch^33^. MemNet leverages AlexNet^22^ as its backbone. AlexNet showed a great performance in image classification task in 2012 and it began a revolution in this computer vision task. MemNet was the first model that utilized deep convolutional neural network for predicting image memorability and it outperformed all of its previous models that were designed to predict memorability scores. MemNet achieves **0.64** rank correlation on memorability scores corrected for false alarms and **0.57** on pure hit rate memorability scores in LaMem dataset. The architecture of MemNet consists of five convolutional and three max-pooling blocks that extract the features for predicting image memorability. Our Pytorch MemNet model reached the same level of performance on LaMem dataset that was mentioned in the original paper ^11^. In addition to MemNet, we also fine-tuned two other models; (**MemVGG** depicted in Fig. 2, and **IncResMem** depicted in Fig. 3) using LaMem dataset to test them on the 10k US Adult Faces Database. *MemVGG* is the memorability predicting model with VGG16 as the backbone architecture. VGG16 is a newer model in comparison to AlexNet and achieves 92.7% top-5 test accuracy on the ImageNet dataset classification while AlexNet achieves 84.6% top-5 test accuracy. This model consists of fifteen convolutional blocks to extract the features followed by three fully connected layers to find the output (image classification on ImageNet^34^ or image memorability on LaMem). We fine-tuned a hybrid VGG16 model which was pre-trained on Places^35^ and ImageNet dataset to predict memorability scores on LaMem dataset. The average performance of this model was **0.63** on 5 test splits of LaMem dataset, however it does not require any complicated preprocessing steps like original AlexNet-based MemNet (see Methods section for more detail). The third model we trained on LaMem dataset is *IncResMem*. Recently Inception models have shown an outstanding performance in different machine vision tasks like image classification. Moreover, it has been shown that training with residual connections accelerates the training of Inception networks significantly. Therefore, we decided to use InceptionResNet^36^ for image memorability prediction. One of the benefit of InceptionResNetv2 model is that it is impervious to noisy labels. Another important feature of residual networks is that they can have very deep architecture while avoiding vanishing gradient problem. We took a pre-trained InceptionResNetv2 model from Keras^37^ that was pre-trained on ImageNet for image classification. This model is deeper in comparison with the previous two models. To obtain better performance, we combined the categories from InceptionResNetV2 with VGG16 features to build the IncResMem model (see Fig. 3). In other words, this model uses both semantic features of the images and also images categories. Stem, Inception and Reduction blocks are identical to the blocks that were introduced by Szegedy et al.^36^. This model achieved **0.646** rank correlation score on the LaMem dataset.

**Figure 4:**
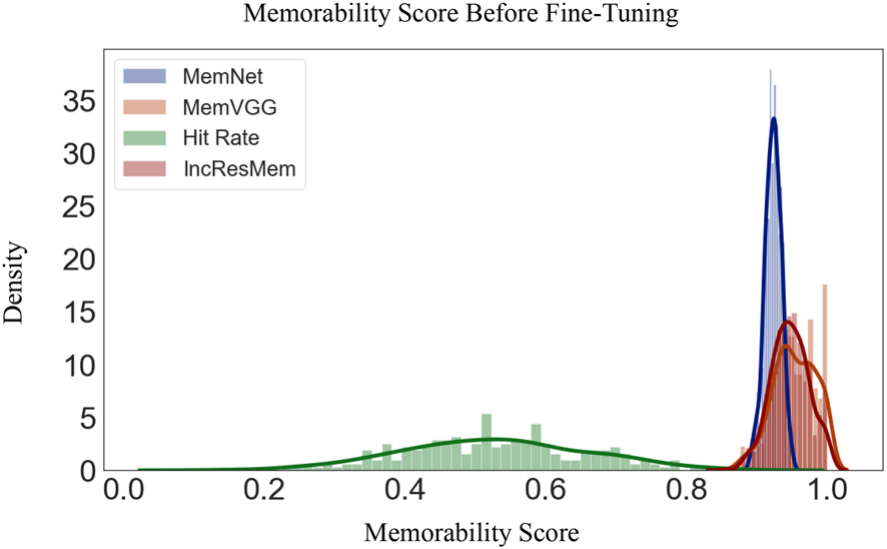
The distribution of ground truth face memorability scores and predictions of MemNet, MemVGG and IncResMem. The mean of the predictions are very high and close to one. The possible reason for this is that these models are trained on LaMem dataset where the images containing people are more memorable compared to images of objects and scenes.

After training these models on LaMem dataset and obtaining valid models for predicting image memorability, we investigated how well they can predict memorability of face images. We observed these models perform poorly in estimating face memorability. The results of this experiment are presented in Table 1. According to this table, the Spearman’s correlation score between predicted memorability scores and ground-truth memorability scores is reported in two categories - hit rate and true hit rate (corrected hit rate). The true hit rate (corrected hit rate) is clacualted by subtracting the false alarm rate from the hit rate. This table illustrates that these models are not able to accurately predict the rank of the memorability scores of face images. The distribution of the predicted face memorability scores of the mentioned three models is shown in Fig. 4. As depicted, these models clearly fail to predict memorability scores for face images. As a result, in the next section we propose and train new models for face memorability prediction.

**Table 1:** Spearman’s correlation score between predicted memorability scores and ground-truth memorability scores is reported in two categories-hit rate and true hit rate (corrected hit rate) obtained by the three proposed Memorability models on 10k US Faces Database.

Model	Hit rate score	True hit rate score
**MemNet**	−0.013	−0.033
**MemVGG**	0.028	0.114
**InceptionResMem**	−0.040	0.013

**Figure 5:**
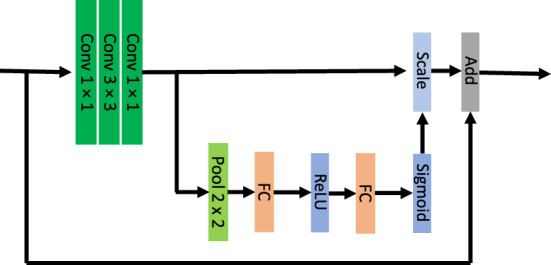
Simple schema of squeeze and excitation block used with identity branch of SENet.

### Memorability models for faces

As we demonstrated in previous section, the models that were trained on the LaMem dataset did not show acceptable performance on predicting face memorability scores. We propose ten new models for predicting face memorability in two groups. The first group consists of the three computational models that are pre-trained on LaMem dataset. We introduced these models (MemNet, MemVGG, and IncResMem) in the previous section. We fine-tuned these models on the 10K US faces dataset for predicting the memorability of faces. The second group includes seven models that are pretrained on VGGFace dataset^32^ and we fine-tuned them on 10k US faces dataset to estimate face memorability scores. Since the memorability dataset for face images is relatively small, the starting point of training process is crucially important and we thought that using pre-trained face models will help us in estimating the face memorability. This seven models include VGG16, ResNet50, SENET50 and all two by two and three by three combinations of features from these three models. We trained all these proposed models both with memorability scores computed by hit rates and the memorability scores corrected by false alarms.

$$\begin{aligned} {\textbf {Hit Rate}} &= \frac{Number\;of\;Hits}{Number\;of\;Hits + Number\;of\;Misses}\\ {\textbf {Corrected Hit Rate}} &= Hit\;Rate - False\;Alarm\;Rate\\ where: \;\;\; False\;Alarm\;Rate &= \frac{Number\;of\;False\;Alarms}{Number\;of\;False\;Alarms + Number\;of\;Correct\;Rejections} \end{aligned}$$

Consistent with Khosla et al.^11^, we observed when the corrected hit rate scores are used, all models outperform the case when hit rate scores are used. That is because false alarm rate introduces noise to memorability scores, therefore, the models perform better when we reduce the noise by correcting for false alarms. Throughout this paper, we refer to the corrected hit rates as true hit rates and the uncorrected ones as hit rates. Moreover, the proposed models of both groups outperformed (see Table 2 and Table 3) the classic MemNet ^11^ (see Table 1) for predicting face memorability. Furthermore, we observed that the models that were based on the pre-trained face models (group 2, see Table 3 ) performed relatively better than memorability networks (group 1, see Table 2) when fine-tuned to predict face memorability. When the models are pre-trained on face images, the weights of the models are optimized to find the face representations that are most useful in the face recognition task. As a result, with only a small dataset of face memorability scores, these models can be further tuned to do a better job of predicting face memorability scores. Comparing the performance of FaceMemVGG in Table 2 to VGG16 in Table 3 evidently shows that the main reason behind the performance difference is the different pretraining schemes. These two models have the same architecture however it is observed that the performance is much better when the model is first pre-trained on the VGGFace database in a face identification task. We should add that Human consistency for the 10k US Faces Database is equal to 0.68 and 0.69 when hit rate and corrected hit rate scores are used, respectively. SENet and SENVGG resulted higher rank correlation compared to the other models. These models employ squeeze and excitation blocks which are beneficial in improving the representational power of the network. These squeeze and excitation blocks are used before summation and also with the identity branch. Simple schema of how these blocks are used has been shown in Fig. 5.

**Table 2:** Memorability scores of the models pre-trained on LaMem dataset and fine-tuned on 10k US face database.

Model	Hit Rate Score	True Hit Rate Score
**FaceMemNet**	**0.424**	**0.543**
**FaceMemVGG**	0.365	0.518
**FaceInceptionResMem**	0.371	0.525

Fine-tuned MemNet model (FaceMemNet) performed better than FaceMemVGG and FaceInceptionResMem in predicting face memorability scores. The best performance is highlighted in bold text.

**Table 3:** Memorability scores of the models pre-trained on face recognition (on VGGfaces database) and fine-tuned on 10K US face database.

Model	Hit Rate Score	True Hit Rate Score
**VGG16**	0.445	0.579
**ResNet50**	0.433	0.607
**SeNet50**	0.448	0.601
**ResVGG**	0.423	0.626
**SenRes**	0.452	0.631
**SenVGG**	**0.468**	0.605
**SenResVGG**	0.445	**0.634**

The results are close to each other, however we can observe that SENRes and SENResVGG show better performance in comparison with other models. Note all these computational models, produce larger Spearman’s rank correlation score when true hit rate scores (corrected scores) are used. The best performance is highlighted in bold text.

### Memorability of oval-shaped and square-shaped faces

**Figure 6:**
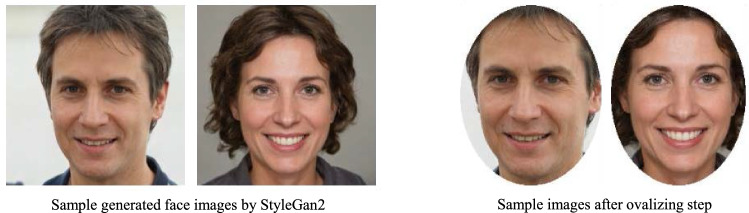
Ovalizing step to make the shape of the synthesized images similar to the dataset that the assessor is trained on.

The 10K US face dataset contains oval-shaped faces with white background. However, it is time and resource-consuming to convert all the face images with the format of this database, whenever we want to predict the face memorability score. Therefore, we decided to test our models on the same set of oval-shaped and square-shaped faces. We utilized StyleGAN2^38^ pretrained on FFHQ dataset ^39^, to generate 8k high-quality and realistic face images. The generated faces from the StyleGAN2 have $1024\times 1024$ resolution in three channels. We changed their size in the pre-processing step, and then calculated their memorability scores with all our models. In order to ovalize these images, we leveraged MTCNN^40^ to detect the coordinates of the faces in the image and then masked an oval on it to make them in the format of the 10k US face database (See Fig. 6). Table 4 shows the Spearman’s rank correlation of predicted memorability scores for the oval-shaped and square-shaped face images. We observe that except for SENVGG, other models that were pre-trained on VGGFace dataset and then fine-tuned with the 10K US face database, result in higher correlation scores compared to the models which are first fine-tuned on LaMem, then fine-tuned on the 10K US face database.

**Table 4:** Rank correlations of predicted memorability scores of square-shaped and oval-shaped face images.

Assessor	Spearman’s correlation
FaceMemNet	0.6976
FaceMemVGG	0.2476
FaceInceptionResNet	0.3687
ResNet50	0.4933
SENet50	0.6217
VGG16	0.6562
ResVGG	0.6793
SENRes	0.6668
SENVGG	0.3520
SENResVGG	0.6609

High correlation score suggests the memorability models perform well on square-shaped faces too.

Table 4 shows that the models with high correlation scores are able to extract face representations even from square-shaped faces and predict their memorability scores. As a result, these models can be utilized in predicting the memorability scores of face images, even without masking and ovalizing them.

## Discussion

Focusing on face images, we showed the existing models used in predicting image memorability fail to predict face memorability scores. We specifically first evaluated MemNet^11^, which is trained on LaMem^11^ dataset. Moreover, we leveraged two other convolutional neural network model architectures (VGG16^29^ and InceptionResNet^36^) and trained them on LaMem dataset for prediction of image memorability. These three models showed great performance in predicting memorability of object, scene and animal images on LaMem Dataset, albeit as we expected, they failed to predict the face memorability scores in 10K US face dataset. The main reason is that these models are trained on LaMem which is a large memorability dataset containing images of objects and scenes. We employed 10K US Face Database ^4^ to fine-tune these three models and observed the rank correlation score significantly increased (see Table 2).

In addition to the three models previously mentioned, we introduced seven new models to estimate face memorability scores. These models are built upon state-of-the-art pre-trained face recognition models^29–31,41^, which we fine-tuned for memorability prediction. Our hypothesis was that these face recognition models, due to their efficiency in extracting facial features, would provide a stronger foundation for predicting face memorability. Furthermore, these new models benefit from simpler preprocessing steps compared to earlier models like MemNet. The backbone architectures of our seven models include VGG16^29^, SENET50^31^, and ResNet50^30^. Each of these has demonstrated excellent performance in various machine learning tasks, particularly in face recognition. Our observations confirmed that models pretrained on facial data are indeed more effective in predicting face memorability. This supports our hypothesis that these models are better at extracting facial features relevant for memorability. Additionally, we considered the possibility that the reduced performance of models trained on the LaMem dataset might be due to the presence of outliers which are unnaturalistic images or those with collage structures. Yet, given that we fine-tuned these models using the 10K US face database, it seems improbable that these few outliers would significantly affect our model’s performance. Future research might delve deeper into examining this aspect.

The 10K US face database primarily contains ovalized face images. However, there may be scenarios where predicting memorability scores for square-shaped face images is desirable. To address this, we tested whether our proposed face memorability prediction models maintain consistent performance on square-shaped faces. It should be noted that in our experiment, a square-shaped face image is not identical to an ovalized one (Fig. 6). Square-shaped images may contain additional elements, such as hairstyles or background features, which could potentially confound the model’s ability to accurately predict face memorability. Yet, our results show that these models can reasonably predict the memorability of square-shaped face images, as indicated by the rank correlation data presented in Table  4.

The proposed models in this work pave the way for predicting memorability of face images in new datasets. Acquiring memorability scores for images requires running large-scale visual memory experiments and crowd sourcing participants usually on online platforms like Amazon Mechanical Turk. These experiments are time-consuming and costly. Having these models will remove this barrier and provides a great opportunity to run future experiments on face memorability.

## Methods

### Deep convolutional neural networks for face memorability prediction

It is common knowledge that deep convolutional neural networks have great power in different machine learning tasks like regression and classification. In this work, we trained two models (MemVGG, IncResMem) on LaMem with the addition of the original MemNet model for predicting image memorability then fine-tuned them on 10k US Adult Faces Database. The backbone of MemNet, MemVGG, and IncResMem are AlexNet, VGG16, and (InceptionResNetv2 and VGG16), respectively. The backbone models of MemNet and MemVGG are pre-trained on both object categories(ImageNet) and scene categories(Places). For the InceptionResMem model, we used pre-trained InceptionResnet model which was pre-trained on Imagenet with addition to a hybrid pre-trained VGG, so we took advantage of both Imagenet and Places classes for predicting memorability scores. The reason behind this choice is that the LaMem dataset is very diverse; It contains objects, scenes, and a combination of objects and scenes. Consequently, to ensure that the model extracts good representation of these images, we chose hybrid models for the backbone of our models. In MemNet, Alexnet features are used, followed by three fully connected layers. The input images were first resized (256,256) pixels with a bi-linear transformation then images were cropped to a (224,224) square shape from the center of the image. All the input images were normalized to the range between 0 and 1. The batch size is 64 in all of the models and Euclidean distance is used as the loss function. The MemVGG model is very similar to MemNet, except the fact that VGG16 is used as the backbone of the model and VGG16 features are used instead of the AlexNet features. In this model, we didn’t need to normalize the input images between 0 and 1. The last model, which we trained on LaMem dataset is IncResMem. Its architecture is shown in Fig. 3. InceptionResNetv2 achieves great performance in image classification task on ImageNet and also can be trained much faster than ResNet models. We combined InceptionResNet categories and VGG16 features, so the layer before the output includes 5096 nodes. The output predicts the memorability of images. There are two separate parallel branch in this network. The size of the input for the VGG16 branch is (224,224) and for the InceptionResnetv2 branch is (299,299).

After training these models on LaMem, we tested them to predict the memorability of the face images. We checked them on the 10k US Adult Face Database and as we expected, these models failed to predict the memorability of faces. As a result, using transfer learning principles, we fine-tuned these three models on US 10k Adult Face Database.

In order to train the models, we split the 10k US Face Database images into train, validation, and test split. We used 80 percent of the data as the training samples and 10 percent of the data for each of the test and validation splits. Again, Euclidean distance was used as the loss function and we set the batch size to 64. Moreover, we leveraged Adam Optimizer to train our models. Due to the large false alarm rate in human face images, we trained our models both with raw memorability scores (computed by hit rate) and corrected memorability scores (considering false alarm rate). We also tried some simple augmentations on the dataset and found, the score of the models will slightly increase if we use simple augmentation like random horizontal flipping ($p=0.5$).

As we mentioned in the Result section, following Khosla et al.^11^, we used linear transformation to set the mean and variance of the predicted memorability samples equal to the train data.

$$\begin{aligned} mean(pred_{i}^{new})&= mean\left[ (pred_{i}^{old} - mean_{predicted}) \times \frac{std_{train}}{std_{predicted}} + mean_{train}\right] \\&= mean\left[ (pred_{i}^{old} - mean_{predicted}) \times \frac{std_{train}}{std_{predicted}}\right] + mean(mean_{train})\\&= mean\left[ (pred_{i}^{old} - mean_{predicted})\right] \times \frac{std_{train}}{std_{predicted}} + mean_{train}\\&= (mean_{predicted} - mean_{predicted}) \times \frac{std_{train}}{std_{predicted}} + mean_{train} = mean_{train} \end{aligned}$$

Equivalently, we can show this similarly for the variances.

Deep convolutional neural networks are capable of extracting the most generic features when they are trained on huge datasets. While we are dealing with face images within a considerably small dataset, we decided to use deep models that are pre-trained on a large face dataset. Therefore, we utilized SeNet50, ResNet50, and VGG16 which are pre-trained on VGGFace dataset^41^ for a face identification task. Face identification is a type of face recognition task where the model weights are optimized to match a human face from a digital image against a database of face identities. VGGFace dataset contains about 2.6 million images within more than 2.6k identities. Images in this dataset have large variations in pose, age, illumination, ethnicity, and profession.

Then, we introduce seven new memorability-predicting models by leveraging SeNet50, ResNet50, and VGG16. All the Face models (SENet, ResNet, and VGG16) were first pre-trained on the VGGFaces database for a face identification task. This database includes about 2.6 million images from more than 2.6k different identities. The architecture of SENet and ResNet are very similar to each other, and the only difference is the existence of Squeeze and Excitation blocks in SENet. However, their architecture is very different from VGG16. VGG16 is a shallower network in comparison with ResNet50 and SENet50. Moreover, all the convolutional filters in VGG16 are $3\times 3$ and max-pooling kernels have a size of $2\times 2$. These models can be divided into three groups. In the first group, we fine-tuned these three models with the 10k US Faces Database to predict face memorability. Features of the last layer from each of the three mentioned models were combined together two by two and formed the second group. Then we fine-tuned them to predict face memorability scores. *ResVGG* was produced by combining the features from the VGG16 network and the ResNet network, *SenVGG* was created from combining the SeNet50 features and the VGG16 features, and a combination of the SeNet50 and ResNet50 features was used to build the *SenRes* model. Finally, we combined features from all these three models and proposed the *SenResVGG* network. We trained all models with a 0.5 chance of horizontal mirroring of the images as an augmentation. We observed that this augmentation helps overcome the over-fitting problem and also increases the rank correlation score.

### Statistical tests

All the reported correlations in Tables 2, 3 and 4 are significant $(p<<0.00001)$. Further, to demonstrate that the correlation scores are higher than any correlations that could be produced from a random distribution, we conducted another experiment and generated two pairs of random vectors of size 8k, for 1000 times and calculated their Spearman’s correlation scores. The average correlation score was about 0 (−0.0003) and the maximum correlation score was 0.028.

### Human participants data

The human participants’ data used in this study are publicly available datasets^4,11^. The participants were provided with an informed consent form which they signed and they were compensated for their time. The protocol was reviewed by the institutional review board at the Massachusetts Institute of Technology. All methods were carried out in accordance with relevant guidelines and regulations.

## Data Availability

The codes and models trained and/or analyzed during the current study are publicly available in https://github.com/mamyou96/FaceMemNet.
